# Retraction: LGD_Net: Capsule network with extreme learning machine for classification of lung diseases using CT scans

**DOI:** 10.1371/journal.pone.0346202

**Published:** 2026-04-08

**Authors:** The PLOS One Editors

The *PLOS One* Editors retract this article [1] due to concerns about potential manipulation of the publication process. These concerns call into question the validity and provenance of the reported results. We regret that the issues were not identified prior to the article’s publication.

In addition, the following concerns were noted:

- All panels of Fig 2 in [1] are similar to Fig 1 in [2] despite being sourced from different datasets.
- The COVID-19 Omicron and pneumonia panels in Fig 2 of [1] are similar to the top two panels in the rows labeled Covid-19, pneumonia, tuberculosis, lung cancer, edema, and COL in Fig 2 of [3] despite representing different conditions.

[2] and [3] were cited in this article [1] as References 36 and 1, respectively.

The first author stated that Fig 2 in [1] presents representative samples from the datasets used in this study. They claimed that the similarities between the images in the three articles may result from the use of similar or related data sources. However, the dataset they mentioned was not used in [2,3]. Concerns remain regarding the source of the data reported in [1] and the discrepancy in the descriptions of the conditions between this article and [2,3].

AHK and MNA did not agree with the retraction. SI responded but expressed neither agreement nor disagreement with the editorial decision. JL either did not respond directly or could not be reached.
